# Simultaneous determination of spirodiclofen, spiromesifen, and spirotetramat and their relevant metabolites in edible fungi using ultra-performance liquid chromatography/tandem mass spectrometry

**DOI:** 10.1038/s41598-021-81013-0

**Published:** 2021-01-15

**Authors:** Fajun Tian, Chengkui Qiao, Caixia Wang, Jing Luo, Linlin Guo, Tao Pang, Jun Li, Ruiping Wang, Rongli Pang, Hanzhong Xie

**Affiliations:** Zhengzhou Fruit Research Institute, Chinese Academy of Agricultural Sciences, Zhengzhou, 450009 China

**Keywords:** Mass spectrometry, Chemical safety

## Abstract

A fast, sensitive, and reliable analytical method was developed and validated for simultaneous identification and quantification of spirodiclofen, spiromesifen, and spirotetramat and their relevant metabolites in edible fungi by ultra-performance liquid chromatography/tandem mass spectrometry (UHPLC–MS/MS). First, sample extraction was done with acetonitrile containing 1% formic acid followed by phase separation with the addition of MgSO_4_:NaOAc. Then, the supernatant was purified by primary secondary amine (PSA), octadecylsilane (C18), and graphitized carbon black (GCB). The linearities of the calibrations for all analytes were excellent (R^2^ ≥ 0.9953). Acceptable recoveries (74.5–106.4%) for all analytes were obtained with good intra- and inter- relative standard deviations of less than 14.5%. The limit of quantification (LOQs) for all analytes was 10 μg kg^−1^. For accurate quantification, matrix-matched calibration curve was applied to normalize the matrix effect. The results indicated that the method was suitable for detecting the three acaricides and their relevant metabolites in edible fungi.

## Introduction

Edible fungi are one of the most important crops worldwide due to its heavy consumption, profitability, high nutritional value, and low calories^1,2^. Chemical analysis shows that edible fungi are rich source of amino acids, fiber, proteins, polysaccharide, chitin, and other substances. These ingredients are capable of preventing to oxidation, tumor, virus, inflammation, lipid accumulation, and cardiovascular disease^3–5^. However, owing to the sealing, humid, and invisible light, the production of edible fungi is seriously affected by various mites, especially, *Siteroptes mesembrinae*, *Caloglyphus mycophagus*, *Tyrophagus putrescentiae*, *Polyphagotarsonemus latus*, etc.^6^. These mites have caused significant economic losses due to high infestation ability. The use of pesticides is the most effective and extensive method to control these mites. Recent reports from China, American, Spain, and other countries have proved that few pesticides have been registered for using on edible fungi and many kinds of illicit pesticides are detected in some commodities^7–9^. Spirocyclic tetronic/tetramic acid (ketoenol) derivatives (spirodiclofen, spiromesifen, and spirotetramat) is the most recently developed class of acaricide. These acaricides acted as an acetyl-coenzyme A carboxylase (ACCase) inhibitor by interfering with lipid biosynthesis^10,11^. Currently, it is extensively used for controlling a wide spectrum of sucking insects in many fruit and vegetable crops in China, such as, red spider in citrus, aphid in cabbage, whitefly in tomato, etc. In order to prevent mites and reduce the economic losses, these acaricides are often used on edible fungi. Therefore, it is important to determine these acaricides residues on edible fungi to ensure the normal foreign trade and food safety.

In recent years, some studies showed that metabolites of some pesticides exhibited equivalent to or higher toxicities and persistence than those of their parent^12,13^. Therefore, several toxicologically significant pesticide residues and metabolites are more and more concerned with humans. Research on environmental pollution and food safety of pesticide residues has become a hot topic. Previous studies showed that spirodiclofen might be a “carcinogenic” pesticide to humans according to USA Environmental Protection Bureau^14^. Spiromesifen has a certain sensitization to the skin. Spiromesifen-alcohol is the major metabolite of spirodiclofen. The data of toxicity is not complete. Spirotetramat is an eye irritant for animals and humans. BYI08330-enol (B-enol), BYI08330-keto-hydroxy (B-keto), BYI08330-mono-hydroxy (B-mono), and BYI08330-enol-glucoside (B-glu) are the major metabolites of spirotetramat. Some studies showed that maternal toxicity was observed at P40 mg/kg bw/day for spirotetramat; further research indicated that male reproductive toxicity is likely caused by the metabolite B-enol in rats^2,15^. Besides, for enforcement and risk assessment purposes in primary crops, spirotetramat, B-enol, B-keto, B-mono, and B-glu are contained in the definition. And some studies also showed that a number of pesticide metabolites are detected in the environment, representing a potential risk for non-target organisms including humans^16^. Therefore, detailed investigations on residue and their metabolism are of great significance. And it is important to develop a specific and sensitive method for the identification and quantification of spirocyclic tetronic/tetramic acid derivatives and their metabolites residues in agricultural samples to maintain food and environmental safety. To our knowledge, there have been many analytical methods to detect single spirocyclic tetronic/tetramic acid derivative and their metabolites residues in agricultural crops^11,15,17–19^. Nonetheless, no peer-reviewed studies have been reported for simultaneous determination of these acaricides in food matrices yet, let alone the multiresidue method determining all three acaricides and their metabolites in edible fungi.

Currently, the maximum residue limits (MRLs) of these acaricides and their metabolites in edible fungi have not been legislated in China, or Japan. However, due to the extensive use of these acaricides, it is necessary to develop a reliable analytical method for determining these acaricides and their metabolites residues to screen their possible illegal use in edible fungi. In addition, since the European Union (EU) has regulated MRLs for these acaricides spirodiclofen (20 μg kg^−1^), spiromesifen (20 μg kg^−1^), spirotetramat and its 4 metabolites (100 μg kg^−1^), it could further protect the export of edible fungi and reduce the economic loss in the international trade for China and other countries. Therefore, the aim of this study was to develop a new method for simultaneous determination of three acaricides and their metabolites in six commonly consumed edible fungi (*Pleurotus ostreatus*, *Agaricus bisporus*, *Lentinus edodes*, *Pleurotus eryngii*, *Hypsizygus marmoreus*, and *Flammulina velutiper*) using a modified QuEChERS (quick, easy, cheap, effective, rugged, and safe) analytical procedure and ultra-performance liquid chromatography/tandem mass spectrometry (UHPLC–MS/MS). For a better extraction and purification effect and higher recoveries, different kinds of extraction solvents and sorbents were investigated for the optimization of the pretreatment method. The development method could be used for the rapid screening of three acaricides and their metabolites in edible fungi.

## Results and discussion

### Optimization of UHPLC–MS/MS conditions

**Figure 1:**
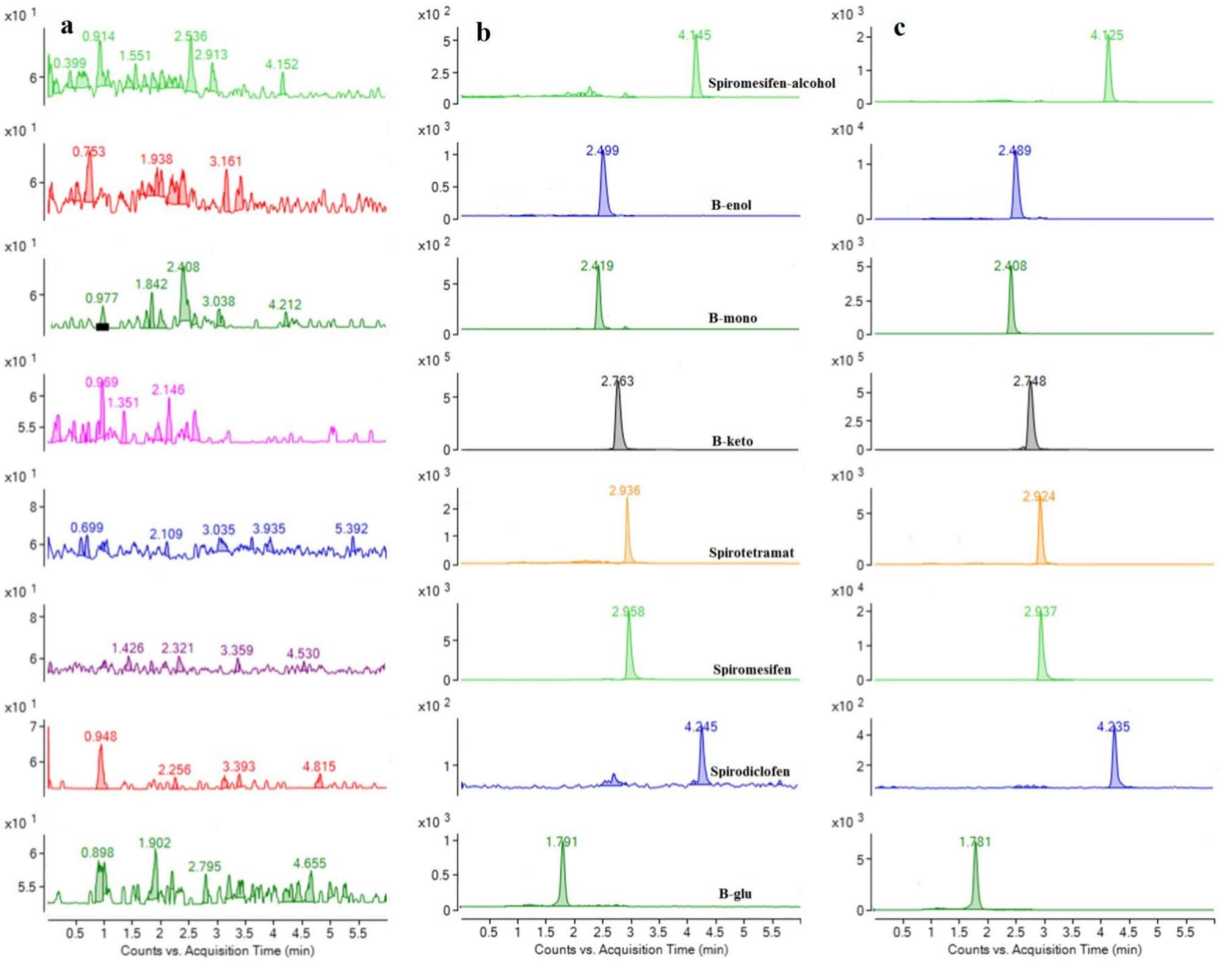
UHPLC–MS/MS chromatograms of spirodiclofen, spiromesifen, and spirotetramat and their relevant metabolites of (**A**) blank *Pleurotus ostreatus* sample, (**B**) *Pleurotus ostreatus* spiked at 100 μg kg^−1^, (**C**) standard (100 μg kg^−1^).

In order to obtain a reliable chromatographic separation of these acaricides and their metabolites with low backpressure and a short analysis time, an Agilent Poroshell 120 EC-C18 column (2.1 × 100 mm, 2.7 μm) was tested. The Poroshell column is composed of 1.7 μm solid silica core with a 0.5 μm porous outer layer. This characteristic makes this column highly efficient for separation of pesticides^20^. Different mobile phase systems (acetonitrile–water, methanol–water, acetonitrile–0.2% formic acid aqueous solution and methanol–0.2% formic acid aqueous solution) were tested and compared. The results showed that the addition of 0.2% formic acid in water enhanced the sensitivity. Therefore, a mobile phase consisting of acetonitrile and water containing 0.2% formic acid aqueous solution was selected as our mobile phase using gradient elution. Under the conditions, the analysis time of these acaricides and their metabolites were less than 6 min, including cleaning and re-equilibration time. And as shown in Fig. 1, no co-eluting peaks were observed near the retention times of the target analytes, indicating the excellent selectivity of the method.

Three acaricides and their metabolites were analyzed in MRM mode. The optimization of MRM parameters including ion transition, fragmentor voltages, and collision voltages, for each target analyte were performed. First, an individual pesticide standard at 0.1 mg L^−1^ was injected into the MS detector to optimize the MRM transitions at a flow rate of 0.3 mL/min. Its precursor ion was obtained in full scan mode in the *m/z* range 50–1000. The molecular ion [M+H]^+^ were selected as the precursor ions for all target compounds. All eight target analytes offered higher precursor ion signal intensities and better fragmentation patterns in positive modes than the negative modes. Next, different fragmentor voltages from 70 to 150 were optimized to acquire the abundance of precursor ions for each compound. In the target ion transitions, the highest intensity was used for quantitation, whereas the second target ion transition was used for identification. At last, the collision energy was also optimized. The precursor ion and two product ions for each analyte were simultaneously monitored and they were used for quantitation and identification. The optimized precursor ions, product ions, fragmentor voltages, and collision voltages for each target compound are listed in Table 1. The results of optimized mass spectrometry parameters are consistent with previous research results^10,11,15,18,19,21^.

**Table 1:** Molecular formula and analytical conditions of the target compounds. ^a^Quantifier. ^b^Qualifier.

Compound	Molecular formula	Ion source	Precursor ion	Fragmentor (V)	Product ion	Collision energy (V)	RT (min)
B-glu	C_24_H_33_NO_8_	ESI^+^	464.3	80	302.2^a^	20	1.78
					270.2^b^	35	
B-mono	C_18_H_25_NO_3_	ESI^+^	304.3	100	254.1^a^	20	2.41
					131.0^b^	30	
B-enol	C_18_H_23_NO_3_	ESI^+^	302.3	80	216.1^a^	20	2.49
					270.1^b^	25	
B-keto	C_18_H_23_NO_4_	ESI^+^	318.2	80	300.2^a^	15	2.75
					268.2^b^	20	
Spirotetramat	C_21_H_27_NO_5_	ESI^+^	374.3	120	330.2^a^	10	2.92
					216.1^b^	40	
Spiromesifen	C_23_H_30_O_4_	ESI^+^	374.4	120	312.4^a^	30	2.94
					267.8^b^	35	
Spiromesifen-alcohol	C_17_H_20_O_3_	ESI^+^	273.2	90	209.0^a^	13	4.12
					206.7^b^	20	
Spirodiclofen	C_21_H_24_Cl_2_O_4_	ESI^+^	411.2	90	313.1^a^	10	4.24
					71.2^b^	12	

### Optimization of extraction and purification procedure

It is of great significance to select a suitable extraction solvent and sorbent for improving extraction efficiency and minimizing matrix interference. Therefore, a range of extraction solvents and sorbents were tested. Recovery experiments were performed to obtain the optimal extraction and purification conditions for each analyte based on recovery value.

**Figure 2:**
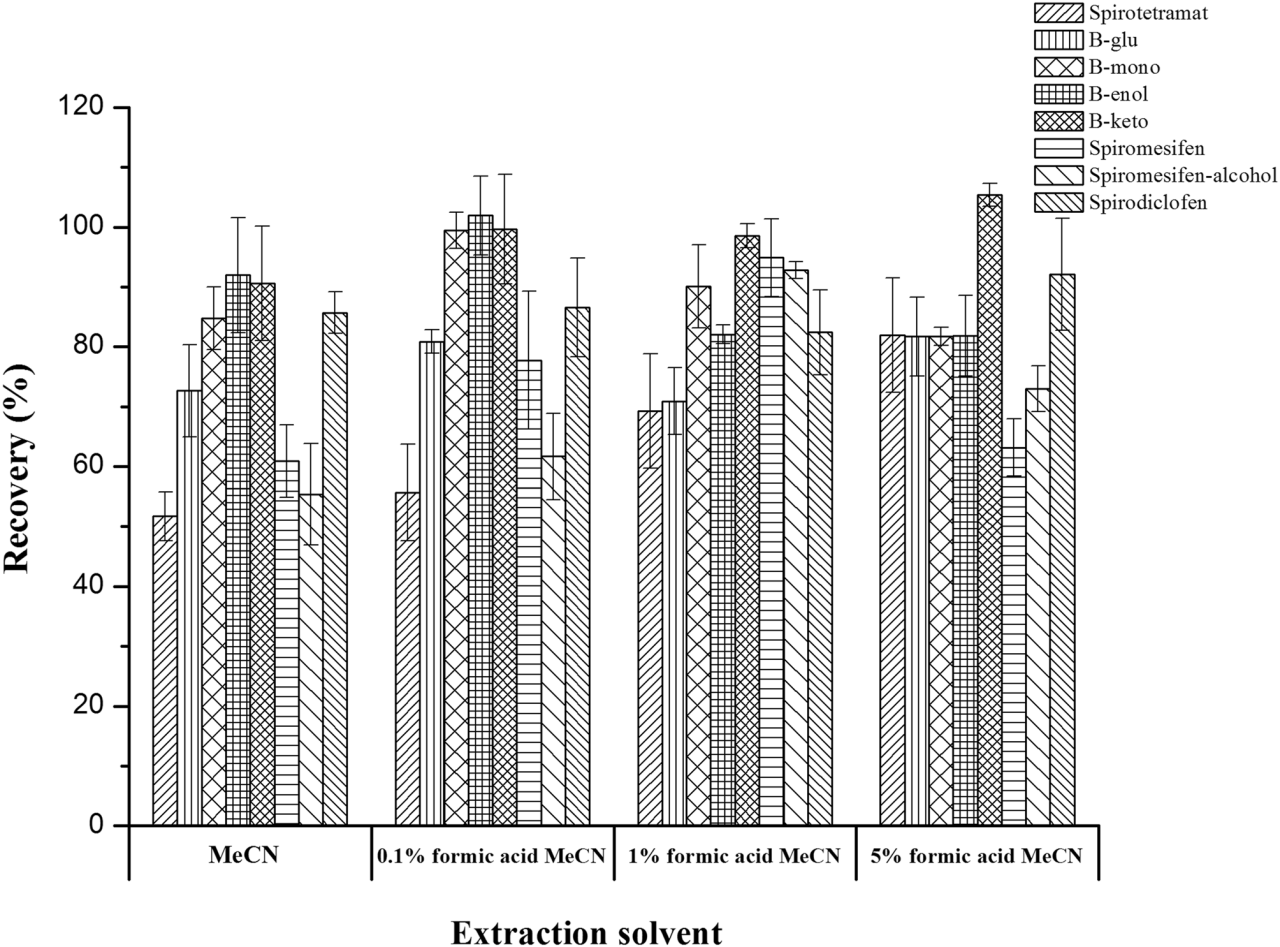
Effect of MeCN containing varying contents of formic acid for targeted compounds in *Pleurotus ostreatus* matrix at the 100 μg kg^−1^ level (n = 3).

Firstly, the extraction solvent was studied. The extraction solvent and the target analytes should have similar polarity. For multi-residue analysis, many studies have demonstrated that MeCN had an appropriate polarity for most analytes and generated higher recoveries and less co-extracted matrix components such as pigments, proteins, lipids, and waxes^11,22–24^. Consequently, MeCN was selected as the extraction solvent in this study. Unfortunately, when using MeCN extraction, the recoveries for B-glu, B-mono, spirotetramat, spiromesifen, and spiromesifen-alcohol were disappointing. In order to improve the recovery of target compounds, we therefore added a certain proportion (0.1%, 1% and 5%) of formic acid to MeCN. As shown in Fig. 2, adding 0.1% formic acid gave an unsatisfactory spiromesifen-alcohol recovery (< 70%). Meanwhile, poor spiromesifen recovery (63.2%) were acquired when 5% formic acid were added. Adding 1% formic acid gave acceptable recoveries (81.8–99.5%) of all the target compounds. Thus, MeCN with 1% formic acid was chosen as the extraction solvent for the target analytes in further study. In addition, anhydrous NaOAc instead of NaCl was used to separate MeCN from water.

**Figure 3:**
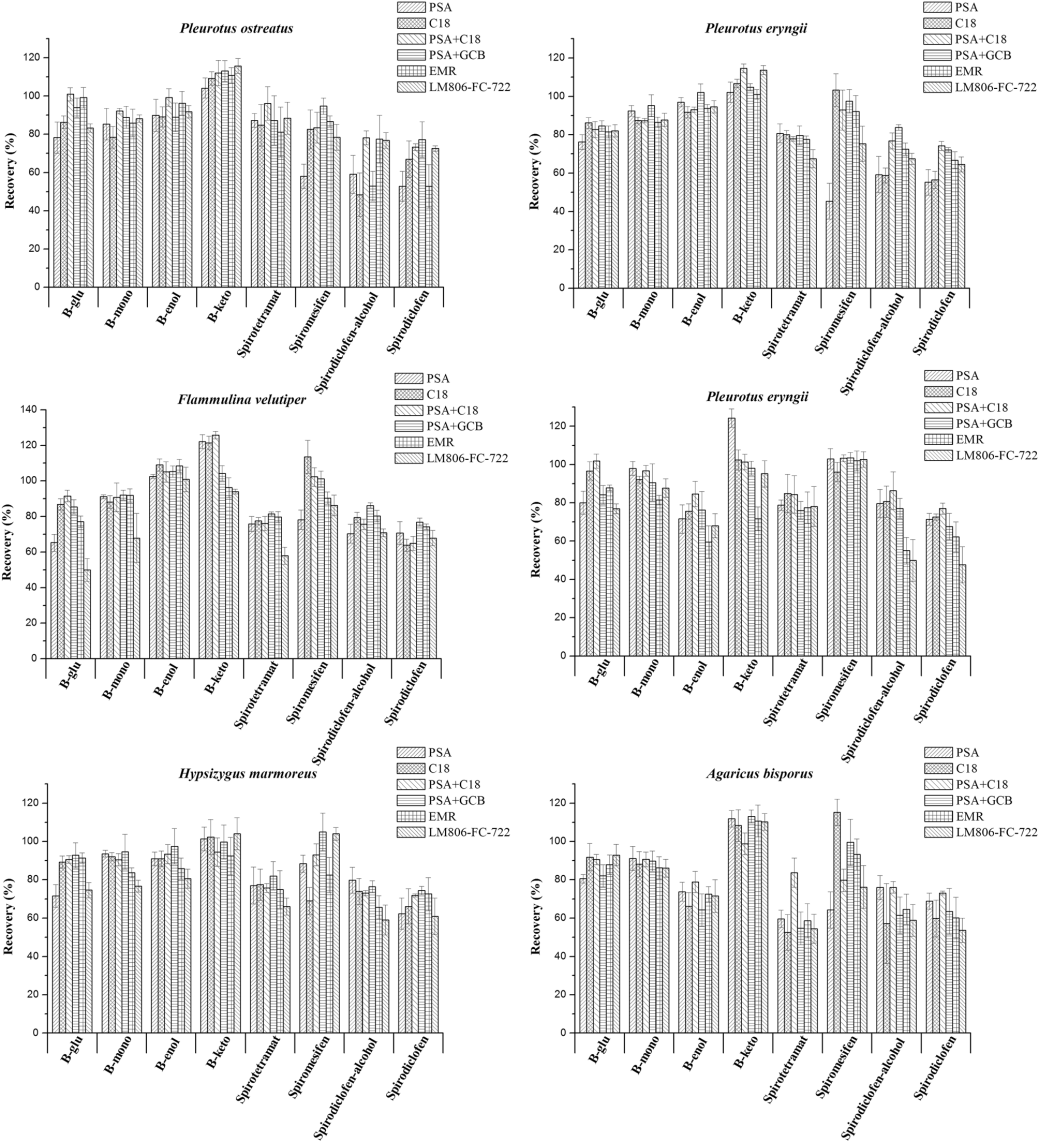
Effect of different sorbents for spirodiclofen, spiromesifen, and spirotetramat and their relevant metabolites in different edible fungi matrix at 100 μg kg^−1^ level (n = 5).

Besides, as complex matrices, edible fungi contained a high amount of compounds, such as amino acids, proteins, polysaccharide, fiber, chitin, and other substances^25^. These compounds may be co-extracted and affect the chromatographic separation. Therefore, the further cleanup procedure is needed to remove these compounds in edible fungi sample. There are numerous cleanup materials for QuEChERS extraction of complex matrices, such as PSA, C18, GCB, EMR-Lipid, and LM806-FC-722^3,15,22^. PSA has a weak anion exchanger that can adsorb various polar matrix components from non-polar samples like fatty acids and sugars, while C18 can be used to remove non-polar and medium-polar interferences from the polar samples. GCB is able to remove pigments, such as carotenoids and chlorophyll^1,23^. Particularly, the EMR-Lipid and CNT are applied to remove the lipid and pigments, respectively^3,26^. In this study, the purification effects of the conventional materials (50 mg PSA + 150 mg MgSO_4_; 50 mg C18 + 150 mg MgSO_4_), their combinations (20 mg PSA + 30 mg C18 + 150 mg MgSO_4_; 25 mg PSA + 2.5 mg GCB + 150 mg MgSO_4_) and the new material EMR-Lipid and LM806-FC-722 were assessed. As shown in Fig. 3, the results demonstrated that there were significant effects exerted by different sorbents on the recoveries of these target compounds in fortified edible fungus samples. The recovery and RSD were both satisfied when 20 mg PSA + 30 mg C18 and LM806-FC-722 were used in the *Pleurotus ostreatus*. Nevertheless, when 20 mg PSA + 30 mg C18 and 25 mg PSA + 2.5 mg GCB as sorbents were used in *Lentinus edodes* and *Hypsizygus marmoreus*, the recoveries of eight target compounds was satisfactory. Meanwhile, 20 mg PSA + 30 mg C18 and EMR-Lipid as sorbents were used in *Flammulina velutiper*, the recoveries was satisfactory. For the *Pleurotus eryngii*, the recovery and RSD were both satisfied when 50 mg C18 and 20 mg PSA + 30 mg C18 were used. And for the *Agaricus bisporus*, only 20 mg PSA + 30 mg C18 as sorbent was used, the recoveries was satisfactory. A possible reason which could account for this was that the polarity of all these target compounds is different, so some compounds could be retained by low-polar or medium-polar sorbents. A study by Tian et al. proved that the significant difference of recoveries of phoxim, chlorpyrifos, and pyridaben were purified by the similar types or amount of sorbents used in our study. Another study performed by Pan et al. also showed a notable difference of recoveries of chlorantraniliprole and cyantraniliprole purified by sorbents like GCB, PSA, etc. Taking the effectiveness and cost of each sorbent into consideration, 20 mg PSA + 30 mg C18 was selected to clean up *Pleurotus ostreatus*, *Pleurotus eryngii*, and *Agaricus bisporus* samples, and 25 mg PSA + 2.5 mg GCB was used to purify the *Lentinus edodes*, *Flammulina velutiper*, and *Hypsizygus marmoreus* samples.

### Method validation

The performance of the developed method was analyzed by evaluating parameters such as specificity, linearity, LOD, LOQ, matrix effects, precision, and accuracy, using matrix spike samples under the above optimization conditions. The results are summarized in Tables 2 and 3.

**Table 2:** Calibration equations, R^2^, LOD, and LOQ of the target compounds in edible fungi. Matrix effect (%) = ((slope matrix/slope solvent) − 1) × 100.

Compound	Matrix	Regression equation	R^2^	Slope ratio (matrix/acetonitrile)	Matrix effect (%)	LOD (μg kg^−1^)	LOQ (μg kg^−1^)	MRL (μg kg^−1^) (EU)
B-glu	Acetonitrile	y = 456.2 x + 3730.4	0.9987	–	–	–	–	–
	*Pleurotus ostreatus*	y = 494.8 x + 456.5	0.9979	1.08	8.46	0.5	10	100
	*Lentinus edodes*	y = 214.0 x + 233.3	0.9994	0.47	− 53.09	1	10	100
	*Flammulina velutiper*	y = 351.3 x – 2660.6	0.9986	0.77	− 22.99	1	10	100
	*Pleurotus eryngii*	y = 433.8 x – 2661.2	0.9992	0.95	− 4.91	1	10	100
	*Hypsizygus marmoreus*	y = 280.2 x – 1181.8	0.9994	0.61	− 38.58	1	10	100
	*Agaricus bisporus*	y = 292.7 x – 1401.1	0.9995	0.65	− 34.85	1	10	100
B-mono	Acetonitrile	y = 1065.1 x – 440.9	0.9968	–	–	–	–	–
	*Pleurotus ostreatus*	y = 462.1 x – 3281.0	0.9983	0.43	− 56.61	1	10	100
	*Lentinus edodes*	y = 482.3 x + 1115.4	0.9998	0.45	− 54.72	1	10	100
	*Flammulina velutiper*	y = 762.6 x + 1339.8	0.9981	0.72	− 28.40	0.5	10	100
	*Pleurotus eryngii*	y = 613.9 x – 909.26	0.9988	0.58	− 42.36	0.5	10	100
	*Hypsizygus marmoreus*	y = 455.1 x – 1382.2	0.9997	0.43	− 57.27	1	10	100
	*Agaricus bisporus*	y = 518.3 x – 903.7	0.9982	0.49	− 51.34	0.5	10	100
B-enol	Acetonitrile	y = 3004.7 x + 21,921.8	0.9991	–	–	–	–	–
	*Pleurotus ostreatus*	y = 1271.5 x – 6344.7	0.9992	0.42	− 57.68	0.5	10	100
	*Lentinus edodes*	y = 2205.3 x + 2972.0	0.9996	0.73	− 26.61	0.1	10	100
	*Flammulina velutiper*	y = 2780.9 x + 4480.2	0.9993	0.93	− 7.45	0.1	10	100
	*Pleurotus eryngii*	y = 1546.7 x – 10,522.8	0.9958	0.51	− 48.52	0.5	10	100
	*Hypsizygus marmoreus*	y = 1743.3 x – 9082.4	0.9995	0.58	− 41.98	0.2	10	100
	*Agaricus bisporus*	y = 1726.6 x – 3202.3	0.9965	0.57	− 42.54	0.1	10	100
B-keto	Acetonitrile	y = 5972.8 x + 152,598.3	0.9998	–	–	–	–	–
	*Pleurotus ostreatus*	y = 3137.0 x + 23,907.5	0.9987	0.53	− 47.48	0.1	10	100
	*Lentinus edodes*	y = 4225.2 x + 116,945.3	0.9984	0.71	− 29.26	0.01	10	100
	*Flammulina velutiper*	y = 4809.9 x + 202,287.8	0.9978	0.81	− 19.47	0.01	10	100
	*Pleurotus eryngii*	y = 3582.6 x + 2643.4	0.9991	0.60	− 40.02	0.05	10	100
	*Hypsizygus marmoreus*	y = 4269.4 x + 76,868.9	0.9965	0.71	− 28.52	0.1	10	100
	*Agaricus bisporus*	y = 3440.8 x + 46,647.3	0.9953	0.58	− 42.39	0.05	10	100
Spirotetramat	Acetonitrile	y = 2412.0 x + 2405.1	0.9985	–	–	–	–	–
	*Pleurotus ostreatus*	y = 1025.0 x – 3644.3	0.9994	0.42	− 57.50	0.5	10	100
	*Lentinus edodes*	y = 1589.1 x + 12,426.0	0.9978	0.66	− 34.12	0.1	10	100
	*Flammulina velutiper*	y = 1410.4 x + 15,794.9	0.9949	0.58	− 41.53	0.2	10	100
	*Pleurotus eryngii*	y = 1935.7 x – 5207.4	0.9994	0.80	− 19.75	0.1	10	100
	*Hypsizygus marmoreus*	y = 1147.2 x – 5124.7	0.9993	0.48	− 52.44	0.5	10	100
	*Agaricus bisporus*	y = 1723.2 x – 2251.5	0.9994	0.71	− 28.56	0.2	10	100
Spiromesifen	Acetonitrile	y = 3730.5 x – 8096.1	0.9993	–	–	–	–	–
	*Pleurotus ostreatus*	y = 1578.2 x – 4739.7	0.9988	0.42	− 57.69	0.5	10	20
	*Lentinus edodes*	y = 2574.3 x + 498.3	0.9993	0.69	− 30.99	0.1	10	20
	*Flammulina velutiper*	y = 1901.6 x + 5996.6	0.9987	0.51	− 49.03	0.1	10	20
	*Pleurotus eryngii*	y = 1836.2 x + 8365.7	0.9972	0.49	− 50.78	0.1	10	20
	*Hypsizygus marmoreus*	y = 2107.9 x – 4802.9	0.9997	0.57	− 43.50	0.1	10	20
	*Agaricus bisporus*	y = 3501.1 x + 3872.7	0.9999	0.94	− 6.15	0.05	10	20
Spiromesifen-alcohol	Acetonitrile	y = 620.5 x + 300.4	0.9999	–	–	–	–	–
	*Pleurotus ostreatus*	y = 276.6 x + 820.3	0.9990	0.45	− 55.42	1	10	–
	*Lentinus edodes*	y = 493.7 x + 5308.1	0.9963	0.80	− 20.43	0.5	10	–
	*Flammulina velutiper*	y = 328.7 x + 2472.3	0.9959	0.53	− 47.03	1	10	–
	*Pleurotus eryngii*	y = 255.7 x + 649.6	0.9991	0.41	− 58.79	1	10	–
	*Hypsizygus marmoreus*	y = 284.6 x – 497.8	0.9987	0.46	− 54.13	2	10	–
	*Agaricus bisporus*	y = 300.6 x – 955.0	0.9984	0.48	− 51.56	2	10	–
Spirodiclofen	Acetonitrile	y = 148.3 x + 362.9	0.9981	–	–	–	–	–
	*Pleurotus ostreatus*	y = 68.7 x + 88.0	0.9991	0.46	− 53.68	2	10	20
	*Lentinus edodes*	y = 117.3 x – 473.2	0.9997	0.79	− 20.90	2	10	20
	*Flammulina velutiper*	y = 72.5 x – 858.7	0.9975	0.49	− 51.11	5	10	20
	*Pleurotus eryngii*	y = 62.9 x – 86.8	0.9985	0.42	− 57.59	5	10	20
	*Hypsizygus marmoreus*	y = 67.0 x + 108.1	0.9988	0.45	− 54.82	2	10	20
	*Agaricus bisporus*	y = 65.6 x – 300.5	0.9995	0.44	− 55.77	5	10	20

**Table 3:** Recoveries (n = 15, %), RSD_r_^a^ and RSD_R_^b^ (%) for target compounds from different matrixes at three spiked levels. ^a^Intra-day (n = 5). ^b^Inter-day (n = 15).

Sorbent	Spiked level (μg kg^−1^)	*Pleurotus ostreatus*			*Lentinus edodes*			*Flammulina velutiper*			*Pleurotus eryngii*			*Hypsizygus marmoreus*			*Agaricus bisporus*		
		10	100	500	10	100	500	10	100	500	10	100	500	10	100	500	10	100	500
		20 mg PSA + 30 mg C18			25 mg PSA + 2.5 mg GCB			25 mg PSA + 2.5 mg GCB			20 mg PSA + 30 mg C18			25 mg PSA + 2.5 mg GCB			20 mg PSA + 30 mg C18		
B-glu	Recovery	93.4	98.2	102.1	78.9	83.8	90.8	91.7	88.7	92.8	97.5	98.7	88.7	86.2	89.7	91.1	85.0	93.6	88.6
	RSD_r_^a^	3.0	4.2	7.6	4.3	3.1	4.6	5.9	4.2	1.9	9.7	3.7	5.1	3.2	1.9	3.1	5.9	2.8	3.6
	RSD_R_^b^	5.7	3.1	9.3	2.5	4.9	2.9	9.4	3.7	2.1	12.4	6.0	6.6	5.4	3.2	4.1	5.2	5.9	4.7
B-mono	Recovery	87.8	93.2	90.8	84.5	92.7	90.8	99.2	95.4	90.6	90.1	93.7	90.2	87.5	91.6	86.8	92.2	93.8	95.3
	RSD_r_^a^	7.0	1.6	2.9	5.2	4.7	4.3	10.6	3.6	3.2	4.8	2.9	5.6	2.1	9.1	5.2	4.0	3.8	5.2
	RSD_R_^b^	9.1	5.4	5.4	7.9	3.9	5.1	13.4	4.8	4.7	6.0	3.6	4.5	3.5	11.2	5.7	6.3	5.2	7.1
B-enol	Recovery	84.9	94.5	84.1	92.1	98.6	90.5	97.8	104.9	93.4	78.9	81.6	87.2	90.4	94.4	92.0	84.9	81.9	90.9
	RSD_r_^a^	1.5	4.7	3.1	3.7	5.7	4.2	7.6	4.2	4.3	2.9	1.7	1.7	5.1	9.2	4.4	9.5	6.5	5.2
	RSD_R_^b^	4.3	7.6	2.7	2.1	8.6	3.2	9.1	7.9	4.7	2.4	6.9	3.6	6.9	8.6	6.7	12.1	8.4	5.8
B-keto	Recovery	96.7	106.4	94.7	88.9	103.2	96.6	91.7	102.5	95.0	95.7	98.2	93.7	91.5	96.7	98.1	103.2	101.8	98.7
	RSD_r_^a^	2.0	6.6	1.9	5.1	6.1	5.9	5.2	5.5	2.1	7.4	4.1	4.1	7.4	8.9	5.0	9.8	5.7	3.4
	RSD_R_^b^	4.1	5.4	2.6	4.2	9.4	7.2	4.9	6.4	3.6	9.8	4.8	8.5	9.2	12.1	5.6	13.9	8.5	5.7
Spirotetramat	Recovery	82.1	93.7	89.5	87.5	83.7	85.2	80.2	85.9	97.5	99.6	94.7	92.5	76.8	79.0	82.5	81.2	86.7	92.1
	RSD_r_^a^	5.2	8.8	3.7	2.2	1.8	5.1	1.7	4.1	4.6	10.7	5.9	4.2	3.5	4.4	5.2	4.5	7.6	3.2
	RSD_R_^b^	4.9	9.2	5.5	2.8	4.8	4.6	4.5	5.6	5.9	14.5	7.0	9.7	4.8	5.1	5.4	3.4	9.5	2.8
Spiromesifen	Recovery	78.5	81.7	84.3	88.4	94.1	91.2	97.9	102.3	91.2	95.4	92.4	89.9	90.2	89.5	95.2	97.5	82.7	88.7
	RSD_r_^a^	1.9	5.1	3.5	2.9	2.4	3.2	5.7	5.8	3.1	3.6	2.8	3.1	5.7	6.0	3.9	8.2	5.8	4.1
	RSD_R_^b^	3.2	7.9	6.2	3.3	3.8	4.7	10.2	7.1	4.5	6.9	5.1	4.0	8.4	7.8	7.6	9.5	6.4	4.4
Spiromesifen-alcohol	Recovery	82.1	76.5	80.2	80.4	85.7	78.9	78.4	84.5	85.9	78.1	81.2	76.4	76.7	78.9	88.5	81.4	79.0	84.6
	RSD_r_^a^	4.5	3.6	3.2	3.5	2.3	1.8	4.2	4.0	3.6	3.2	5.2	3.0	2.7	3.3	3.6	4.3	3.1	5.1
	RSD_R_^b^	5.9	2.1	4.9	4.4	6.6	3.3	3.7	5.5	4.0	1.5	7.0	2.8	2.6	4.5	5.1	6.4	4.5	5.9
Spirodiclofen	Recovery	78.1	74.5	81.4	75.9	77.6	82.1	82.7	78.9	83.1	79.8	74.8	77.8	80.1	76.4	82.4	83.6	76.1	81.4
	RSD_r_^a^	4.1	1.6	3.2	2.1	1.2	3.9	5.2	3.1	1.3	1.9	1.5	2.0	4.4	2.2	4.0	4.7	2.9	2.3
	RSD_R_^b^	5.2	2.1	5.8	3.0	2.7	4.2	5.7	2.4	4.6	4.7	2.6	3.5	5.1	3.4	3.8	3.9	4.2	4.1

#### Linearity, LOD, LOQ, and matrix effect

For all target compounds, the good linearities with correlation coefficients (R^2^) exceeding 0.9953 were obtained in all matrices, ranging from 5.0 to 500 μg kg^−1^. The LODs for all target compounds were 0.010–5 μg kg^−1^. The LOQs for these target compounds were validated with acceptable accuracy at the lowest spiked concentration of 10 μg kg^−1^ in all edible fungi matrices. The LOQs for eight target compounds was below the maximum residue limit (MRLs) recommended by the EU in edible fungi (spiromesifen and spirodiclofen, 20 μg kg^−1^ in edible fungi; spirotetramat and its four metabolites, 100 μg kg^−1^).

In pesticide residues analysis, matrix effect was reported to be problematic using GC–MS/MS and LC–MS/MS^23,27^. Matrix effect is the comprehensive effect of all components in the sample except the target compounds on the measurement, which is considered to suppress or enhance analyte signals due to the co-elution of matrix components^28–31^. It can play an important role in the quality of quantitative data generated by the method. Therefore, the matrix effect was researched. A result higher than ± 10% (slope ratio of higher than 1.1 or lower than 0.9) indicated that signal enhancement or suppression were observed in the matrix. It also meant that there was a significant matrix effect and this could not be ignored. When the slope ratio was within 0.9–1.1, the matrix effect can be considered negligible^22,24^. As shown in Table 2, the obvious signal suppression or enhancement differences were observed for all target analytes in six edible fungi matrices as the slope ratios of matrix/acetonitrile were in the range of 0.41–1.08. All the analytes showed a signal suppression effect (− 57.69% ≤ ME ≤ − 19.47%) in all matrices except for B-glu in *Pleurotus ostreatus* (ME = 8.46%) and *Pleurotus eryngii* (ME = − 4.91%), B-enol in *Flammulina velutiper* (ME = − 7.45%), and spiromesifen in *Agaricus bisporus* (ME = − 6.15%). These matrix effects could be ignored. The above results also indicated that matrix effect mostly in the form of ion suppression still existed in all compounds despite the inclusion of a cleanup step. The results from other researchers were consistent with ours. For example, some studies also reported that dinotefuran, pyriproxyfen, avermectin, and difubenzuron in edible fungi had strong suppression^3,7,9^. This may be related to the edible fungi matrices. The results also showed that the matrix effect depended on the analyte. Therefore, the matrix effect was both matrix dependent and compound dependent. However, the actual reason and mechanism of these matrix effects are not fully understood. Therefore, the matrix-matched calibration curve was used to eliminate the matrix effect and obtain more accurate results for each target compound in the samples.

#### Precision and accuracy

Recoveries and RSD of these target compounds studies were done by spiking of matrix spike samples with different concentrations (10, 100, and 500 μg kg^−1^). Five replicates were employed at each level of fortification for all the target compounds on each of the three distinct days. The recoveries were calculated using the six point matrix-matched calibration standards. The precision of method was expressed as the RSD for repeatability (RSD_r_) and reproducibility (RSD_R_). As shown in Table 3, good recoveries (74.5–106.4%) for all the target compounds with all RSD values below 14.5% at all levels of fortification were obtained. Furthermore, the RSD_r_ and RSD_R_ for all target compounds ranged from 1.2 to 10.6% and from 1.5 to 14.5% in all matrices, respectively. The mean recoveries for B-glu were 78.9–102.1% with 1.9–12.4% RSDs, 84.5–99.2% with 1.6–13.4% RSDs for B-mono, 78.9–104.9% with 1.5–12.1% RSDs for B-enol, and 88.9–106.4% with 1.9–13.9% RSDs for B-keto, while they were from 76.8 to 99.6% with 1.7–14.5% for spirotetramat, from 78.5 to 102.3% with 1.9–10.2% for spiromesifen, and from 76.4 to 88.5% with 1.5–7.0% for spiromesifen-alcohol, finally, for spirodiclofen, the mean recoveries were in the range of 74.5–83.6% with RSDs of 1.2–5.8%. The results of the recovery studies indicated that the proposed method exhibited good accuracy and precision for the three acaricides and their metabolites residue analysis in edible fungi. Overall, it also indicated that the proposed method was reliable and suitable for the routine analysis of the three acaricides and their metabolites residues in edible fungi.

### Application to real samples

The proposed method (QuEChERS-UHPLC–MS/MS) in this study was applied to the real edible fungi samples. These samples were obtained from local markets in Zhengzhou, Henan Province (China). These samples contained *Pleurotus ostreatus*, *Agaricus bisporus*, *Lentinus edodes*, *Pleurotus eryngii*, *Hypsizygus marmoreus*, and *Flammulina velutiper*, and each matrix purchased 10 samples. These samples were treated with the sample preparation method described in “Materials and methods”. Three *Pleurotus ostreatus* samples and one *Lentinus edodes* sample were positive, suggesting that they contained spirotetramat in the range of 21–48 μg kg^−1^. The residue of spirodiclofen was also detected in two *Lentinus edodes* samples, which was lower than LOQ. Although spirotetramat has determined in some edible fungi samples, it does not pose a threat to the consumer since they are lower than the MRLs established by the EU (100 μg kg^−1^). The results indicated that the ongoing performance and ruggedness of the proposed method were very satisfactory.

In this study, a simple, sensitive, rapid, and reliable pretreatment method was developed and validated for the determination of spirodiclofen, spiromesifen, and spirotetramat and their relevant metabolites in edible fungi matrices using UHPLC–MS/MS. Samples were extracted by MeCN, containing 1% (v/v) of formic acid and then cleaned up by 20 mg PSA + 30 mg C18 for *Pleurotus ostreatus*, *Pleurotus eryngii*, and *Agaricus bisporus* samples, and 25 mg PSA + 2.5 mg GCB for *Lentinus edodes*, *Flammulina velutiper*, and *Hypsizygus marmoreus* samples. Instrumental analysis employing UHPLC–MS/MS was operated in ESI + mode. The eight target analytes were successfully separated within 6 min. The method revealed satisfactory recoveries, linearities, precision and accuracy, and low LODs and LOQs. Therefore, the proposed method could be a useful means for monitoring three acaricides and their metabolites’ residues in edible fungi samples to ensure food safety.

## Materials and methods

### Materials and reagents

Certified standards of spirodiclofen (purity 99.9%), spiromesifen (purity 99.2%), spirodiclofen-alcohol (purity 98.0%), spirotetramat (purity 98.6%), and the four metabolites of spirotetramat (B-enol (purity 99.9%), B-keto (purity 99.8%), B-mono (purity 99.7%), and B-glu (purity 97.9%)) were purchased from Alta Scientific Co., Ltd. (Tianjin, China). HPLC grade acetonitrile (MeCN) and formic acid were supplied from Honeywell International Inc. (New Jersey, USA). Anhydrous magnesium sulfate (MgSO_4_) and sodium acetate (NaOAc) obtained from Sinopharm Chemical Reagent Ltd. (Shanghai, China). The water used was purified with Millipore purification system (Bedford, MA, USA). Primary secondary amine (PSA), octadecylsilane (C18) and graphitized carbon black (GCB) of mesh size of 40 μm were procured from Agela Technologies Inc. (Beijing, China), and enhanced matrix removal-lipid (EMR-Lipid) was also purchased from Agela Technologies Inc. Dispersive solid-phase cleanup sorbent multiplug filters (LM806-FC-722) contained carbon nanotubes (CNT) and PSA were purchased from Lumiere Tech. Ltd. (Beijing, China).

Individual standard pesticides and metabolites stock solutions (1000 mg L^−1^) were dissolved in pure MeCN. The standard multi-component solution was prepared by mixing appropriate volumes of each standard solutions to obtain a concentration of 100 mg L^−1^, and the resulting solution was utilized for spiking edible fungi samples to prepare the matrix-matched calibration. Calibration standards of 5, 10, 50, 100, 200, and 500 μg L^−1^ were prepared from above mixture solution by suitable dilutions with MeCN. Likewise, the matrix-matched calibration standards (5, 10, 50, 100, 200, and 500 μg L^−1^) were prepared by adding blank sample extracts. For the preparation of matrix-matched standard, appropriate volumes of work standard solution was firstly dried under nitrogen and then redissolved by 1 mL blank sample extract. The stock solutions did not degrade for at least three months. All the solutions were stored at − 20 °C in the dark.

### Instrumental parameters

Chromatographic separation of three cyclicketoenole acaricides and their metabolites was carried out on an Agilent 1290 Infinity UHPLC coupled to an Agilent 6460A triple quadrupole mass spectrometer equipped with an electrospray ionization (ESI) source (Agilent Technologies, Inc., Santa Clara, CA, USA) with an Agilent Poroshell 120 EC-C18 column (2.1 × 100 mm, 2.7 μm). The column temperature was maintained at 35 °C during the experiments. The flow rate was 0.3 mL min^−1^ and the injection (5 μL) was conducted by using an autosampler. The mobile phases A and B were 0.2% (v/v) formic acid and MeCN, respectively, operating under gradient elution. Elution was carried out as follows: 0–1 min, 90–60% A; 1–2 min, 60–10% A; 2–5 min, 10% A; 5–5.1 min, 10–90% A; 5.1–6 min, 90% A. The total run time was 6 min and all the analytes were eluted within 4.3 min. The temperature of sample vial holder was kept at 5 °C. Under these conditions the retention times for B-glu, B-mono, B-enol, B-keto, spirotetramat, spiromesifen, spiromesifen-alcohol, and spirodiclofen were found at 1.78, 2.41, 2.49, 2.75, 2.92, 2.94, 4.12, and 4.24 min, respectively.

The mass spectrometer was operated in positive ESI mode and multiple reactions monitoring (MRM) acquisition mode to determine the above-mentioned acaricides and their metabolites. The MS conditions were typically as follows: drying gas flow, 8 L min^−1^ and drying gas temperature 350 °C; capillary voltage 4000 V; sheath gas flow, 12 L min^−1^ and sheath gas temperature 350 °C; nebulizer pressure, 35 psi. Nitrogen served as the nebulizer and collision gas. All MS parameters (precursor ions, product ions, fragmentor voltage, and collision energy) were optimized individually for each target compound by flow injection analysis at the concentration of 0.1 mg L^−1^ and were shown in Table 1. Agilent MassHunter Workstation software (version B.03.01) was used to acquire and process data in the method development.

### Sample preparation

Fresh samples of six edible fungi without pesticides were achieved from the experimental base in Fuji Town, Qi Country, Henan Province, China. The three pesticides have not been used. About 900 g samples were homogenized by Ultra-Turrax homogenizer (IKA-Werke, Staufen, Germany). Then, they were stored at less than − 18 °C until analyses in the dark. The edible fungi samples were only homogenized before weighing.

A 10 g of the homogenized fortified sample was weighed in a 50 mL centrifuge tube. 10 mL of MeCN, containing 1% (v/v) of formic acid, were then added and the mixture was shaken for 10 min. Afterwards, anhydrous MgSO_4_ (4 g) and anhydrous NaOAc (1 g) were added, and the tube was sealed and shaken vigorously for 5 min to provide well-defined phase separation after 5 min of centrifugation at 2880*g*. During the cleanup step, 1.5 mL of upper MeCN layer was transferred into a 2.0 mL centrifuge tube containing an amount of different sorbent (20 mg PSA + 30 mg C18 for *Pleurotus ostreatus*, *Pleurotus eryngii*, and *Agaricus bisporus* samples; and 25 mg PSA + 2.5 mg GCB for *Lentinus edodes*, *Flammulina velutiper*, and *Hypsizygus marmoreus* samples) and 150 mg anhydrous MgSO_4_. The tubes were vortexed vigorously for 5 min and centrifuged for 5 min at 2400*g*. Then, the MeCN layer obtained by centrifugation was filtered through a 0.22 μm nylon syringe filters and transferred into an autosampler vial for UHPLC–MS/MS analysis.

For the EMR-Lipid cleanup, the extraction steps are the same as mentioned above. 5 mL Milli-Q water was added into EMR-Lipid tube to activate the sorbent. Next, a 5 mL upper layer (MeCN) was added into the tube. Then, the tube was vortexed for 5 min and centrifuged for 5 min at 3913* g*. Afterwards, a 5 mL upper layer was transferred into the EMR-Polish tube that containing 400 mg of sodium chloride and 1600 mg of MgSO_4_. The tube was vortexed for 5 min and centrifuged for 3 min at 2811*g*. Finally, a 1 mL upper layer filtered through a 0.22 μm nylon syringe filter before instrumental analysis.

### Validation of the analytical procedure

Method trueness and precision were evaluated by recovery experiments using blank matrices of the six studied edible fungi spiked at three concentration levels, 10, 100, and 500 μg kg^−1^, with five replicates for each level on three different days. Recovery results of spiked samples were used as the evaluation of trueness, and the intra-day and inter-day relative standard deviations (RSD) were used as the evaluation of precision. The accurate quantification of the analytes was achieved by using matrix-matched calibration curves which were prepared by spiking an aliquot of the blank extract with the desired amount of standard solution. The linearity of calibration curves for all the target compounds was evaluated both in solvent and matrix in a concentration range of 5–500 μg L^−1^. Matrix effects were calculated as follows: matrix effect (%) = (slope of calibration curves in matrix − slope of calibration curves in solvent)/slope of calibration curves in solvent (× 100%). Blank samples of each matrix were analyzed to identify the absence of interfering species during the retention time of the analytes. The limit of quantitation (LOQ) for each analyte was defined as the lowest validated spiked level in different matrices meeting the requirements of a recovery within the range 70–120% and the RSD lower than 20%.
